# Volatile Composition of Essential Oils from Different Aromatic Herbs Grown in Mediterranean Regions of Spain

**DOI:** 10.3390/foods5020041

**Published:** 2016-05-25

**Authors:** Hussein El-Zaeddi, Juan Martínez-Tomé, Ángel Calín-Sánchez, Francisco Burló, Ángel A. Carbonell-Barrachina

**Affiliations:** 1 Research Group “Food Quality and Safety”, Department of Agro-Food Technology, Miguel Hernández University, Carretera de Beniel, km 3.2, 03312-Orihuela, Alicante, Spain; elzaeddi@gmail.com (H.E.Z.); francisco.burlo@umh.es (F.B.); angel.carbonell@umh.es (A.A.C.B.); 2 Department of Plant Sciences and Microbiology, Miguel Hernández University, Carretera de Beniel, km 3.2, 03312-Orihuela, Alicante, Spain; juan.martinez@umh.es (J.M.T.)

**Keywords:** dill, parsley, coriander, mint, GC-MS, descriptive sensory analysis

## Abstract

Volatile composition of essential oils from dill, parsley, coriander, and mint were investigated at different harvest dates to determine the most suitable harvest time for each these herbs. Hydrodistillation (HD), using a Deryng system, was used for isolating the essential oils. Isolation and identification of the volatile compounds were performed using gas chromatography-mass spectrometry (GC-MS) instrument. The results of gas chromatography-flame ionization detector (GC-FID) analysis (quantification) showed that the main components in the essential oil of dill shoots were α-phellandrene, dill ether, and β-phellandrene, and the optimal harvest date was D2 (second harvest, fourth week of February 2015). For parsley shoots, the main compounds were 1,3,8-*p*-menthatriene, β-phellandrene, and P1 (first harvest, third week of November 2014) was the sample with the highest essential oil. For coriander, the main compounds were *E*-2-dodecenal, dodecanal, and octane and the highest contents were found at C2 (second harvest, 5 February 2015); while, the main two components of mint essential oil were carvone and limonene, and the highest contents were found at M1 (first harvest, second week of December 2014). The present study was the first one reporting data on descriptive sensory analysis of aromatic herbs at this optimal harvest date according to the content of volatile compounds of their essential oils.

## 1. Introduction

The production of aromatic herbs, such as oregano, marjoram, rosemary, thyme, lavender, or peppermint, is a rising sector in the Mediterranean countries. These plants have many applications, such as ornamental cropping, perfumery, or food and pharmaceutical industries; these later applications are linked to the beneficial health effects of their essential oils, which have antimicrobial, antifungal, insecticidal, and antioxidant properties [1,2].

Dill (*Anethum graveolens* L.) is an important aromatic herb, which is used as flavoring and seasoning of various foods, such as salads, sauces, soups, sea foods, and especially pickled vegetables [3]. Parsley (*Petroselinum crispum*) and coriander (*Coriandrum sativum*) are two culinary herbs commonly used to enhance the flavor of many dishes of the cuisines of China, Mexico, South America, India, and South East Asia [4]. Peppermint (*Mentha piperita* L.) is a famous aromatic and medicinal herb used in traditional and folk medicines in the world for its antimicrobial and antioxidant properties [5].

In addition, culinary herbal extracts and essential oils have become increasingly popular as alternative sources of natural preservative agents, largely because herbs are widely cultivated, effective, and safe for consumption [5]. Essential oils are extracted from various aromatic plants generally localized in temperate to warm countries, such as the Mediterranean countries [1]. Peppermint (*M. piperita* L.) essential oils have been obtained by steam distillation in a Clevenger-type apparatus [5], while microwave extraction technique of mint essential oil was used by Costa *et al.* [6]. Besides, Huopalahti and Linko [3] isolated aroma compounds of dill (*Anethum graveolens* L.) by using solvent extraction technique.

It is well-known that the presence of essential oils and their composition determine the specific aroma of plants and the flavor of the resulting condiments [7,8]. The main chemical families present in aromatic herbs are: *monoterpenes*, *monoterpenoids*, and *phenylpropanoids*. In lower amount alcohols, *sesquiterpenes*, *sesquiterpenoids*, *aldehydes*, and *esters* were also found [9,10,11,12]. The composition and concentrations of essential oils from aromatic herbs depend on many factors, including geographical source, climatic and soil conditions, stage of vegetative cycle, seasonal variation, *etc.* [13,14,15].

Therefore, the aim of this study was to analyze the volatile composition of the essential oils and the sensory quality of different aromatic herbs (parsley, dill, mint, and coriander) grown in different Mediterranean regions of Spain in order to stablish the best harvest time according to the highest content of essential oils and the optimum sensory quality.

## 2. Materials and Methods

### 2.1. Plant Material

Four aromatic herbs (dill, parsley, coriander, and mint) were grown under conventional agricultural practices and conditions according to the recommendations of farmers located at Mediterranean regions of Spain according to their experience. These conditions are described as follows:

Dill seeds (*Anethum graveolens* L. Cv. ELLA) were sown on the 7 September 2014 in expanded polystyrene (EPS) trays and placed in a greenhouse located at Santomera (Murcia, Spain) until 4 October 2014. Then, plantlets were transplanted into a commercial orchard placed at Sucina (Murcia, Spain). Dill samples at the commercial stage were harvested at two different dates within the same plant on the 26 November 2014 (D1: 80 days after sowing) and 28 February 2015 (D2: 174 days after sowing).

Parsley seeds (*Petroselinum crispum* Cv. Gigante Italiano Darkness) were sown on the 2 September 2014 in expanded polystyrene (EPS) trays and placed in a greenhouse located at Santomera (Murcia, Spain) until 24 September 2014. Then, plantlets were transplanted into a commercial orchard placed at Sucina (Murcia, Spain). Parsley samples at commercial stage were harvested at three different dates within the same plant on the 19 November 2014 (P1: 78 days after sowing), 5 January 2015 (P2: 144 days after sowing), and 25 February 2015 (P3: 175 days after sowing).

Coriander seeds (*Coriander sativum* Cv. MARINO) were directly sown on the commercial orchard placed at Sucina (Murcia, Spain). Coriander has only one harvest for each plant and in these regions three crops per year are sown, grown, and harvested. The first crop (C1) was planted on 30 September and harvested on 19 November 2014; the second crop (C2) was planted on 22 October 2014 and harvested on 5 February 2015; and, the third crop (C3) was planted on 29 December and harvested on 25 February 2015.

Mint cuttings (*Mentha piperita* L.) of 5–7 cm were sown on 7 August 2014 in polyethylene trays and placed in a greenhouse located at Santomera (Murcia, Spain) until 6 October 2014. Then, plantlets were transplanted to a commercial orchard placed at Sucina (Murcia, Spain). Mint samples at commercial stages were harvested at two different dates from the same plant on the 11 December 2014 (M1: 133 days after sowing) and 5 February 2015 (M2: 189 days after sowing).

Aromatic herbs were grown using high-frequency drip irrigation systems. The water contribution was carried out using polyethylene pipes of 16 mm of diameter. The emitters were adjusted at 32 cm of distance with a total flow of 1.6 L·h^−1^. The total volume of water for each crop was as following: 3208 m^3^·ha^−1^ for dill, 3849 m^3^·ha^−1^ for parsley, 3445 m^3^·ha^−1^ for coriander, and 2566 m^3^·ha^−1^ for mint.

The irrigation water was of good quality, highlighting its slightly basic pH (7.91), and its proper electrical conductivity (1.26 mS·cm^−1^), which is suitable for aromatic herbs crops. Soil was uniformly silty-loam in texture, with a low content in organic matter (1.22%), medium salinity conditions (3.35 mS·cm^−1^), and good levels of sulfates (37.83 meq·L^−1^) for aromatic herb development.

Along the development of crops, fertilization was carried out with a total amount of N of 130 kg·ha^−1^, P (P_2_O_5_) of 60 kg·ha^−1^, and K (K_2_O) of 160 kg·ha^−1^ for dill development. For parsley, the total amount of fertilizers was 300 kg·N·ha^−1^, 190 kg·P·ha^−1^ (P_2_O_5_), and 350 kg·K·ha^−1^ (K_2_O). Regarding coriander, the fertilization was carried out with a total amount N of 275 kg·ha^−1^, P (P_2_O_5_) of 170 kg·ha^−1^, and K (K_2_O) of 310 kg·ha^−1^. Finally, the total amount of fertilizers was 200 kg·N·ha^−1^, 130 kg·P·ha^−1^ (P_2_O_5_), and 230 kg·K·ha^−1^ (K_2_O).

### 2.2. Extraction of Essential Oils

Hydrodistillation (HD), using a Deryng system (the Polish version of the Clevenger apparatus), was used for isolating the essential oil in fresh herbs (dill, parsley, coriander, and mint). About 15.0 g of freshly chopped herbs shoots (aerial part of the plant, including stems and leaves) were put in a 500 mL round bottom flask, together with 1.0 g sodium chloride (NaCl), 150 mL of distilled water, and 50 µL of benzyl acetate as an internal standard (987 mg·L^−1^). After the mixture started boiling, heating was maintained for 1 h. A cold refrigerant was used to condense the vapors, and 1 mL of cyclohexane was added to the Deryng apparatus at the beginning of the hydrodistillation process to retain the essential oil distilled from the samples of herbs shoots. After 60 min of extraction, the solvent, enriched with the volatile compounds, was transferred into a 2.5 mL vial, after drying it over anhydrous sodium sulfate (Na_2_SO_4_), and kept at −15 °C until the gas chromatography-mass spectrometry (GC-MS) analyses were conducted. The extractions were conducted in triplicate.

### 2.3. Chromatographic Analyses

Analysis and identification of the volatile compounds were performed using a Shimadzu GC-17A gas chromatograph coupled with a Shimadzu QP-5050A mass spectrometer detector (Shimadzu Corporation, Kyoto, Japan). The GC-MS system was equipped with a TRACSIL Meta.X5 (95% dimethylpolysiloxane and 5% diphenylpolysiloxane) column (60 m × 0.25 mm, 0.25 μm film thickness; Teknokroma S. Coop. C. Ltd, Barcelona, Spain). Analyses were carried out using helium as carrier gas at a column flow rate of 0.3 mL·min^−1^ and a total flow of 3.9 mL·min^−1^ in a split ratio of 1:11 and the following program: (a) 80 °C for 0 min; (b) increase of 3 °C·min^−1^ from 80 °C to 210 °C and hold for 1 min; (c) increase of 25 °C·min^−1^ from 210 °C to 300 °C and hold for 3 min. The temperatures of the injector and detector were 230 °C and 300 °C, respectively.

All compounds were identified using three different analytical methods: (1) comparison of experimental retention indexes (RI) with those of the literature; (2) GC-MS retention times (authentic standards of “all” compounds reported in Table 1, Table 2, Table 3, Table 4 and Table 5 were used for identification purposes); and, (3) mass spectra (authentic chemicals and NIST05 spectral library collection). Only fully identified compounds have been reported in this study.

The semi-quantification of the volatile compounds was performed on a gas chromatograph, Shimadzu 2010, with a flame ionization detector (FID). The column and chromatographic conditions were those previously reported for the GC-MS analysis. The injector temperature was 200 °C and nitrogen was used as carrier gas (1 mL·min^−1^). The quantification was obtained from electronic integration measurements using flame ionization detection (FID). Benzyl acetate (1000 mg·L^−1^) was added as internal standard at the beginning of the distillation procedure to simulate the behavior of volatile compounds; this chemical was used as an internal standard after checking that it was absent in herbs, it separates well from other volatiles, it possesses similar FID and MS response factors to most of the volatiles in the aromatic herb essential oil, it is stable at high temperatures, and does not react with water. Calibration curves were performed with the following compounds (Sigma-Aldrich, Madrid, Spain) as representative of each chemical family: α-phellandrene (monoterpenes), α-terpineol (terpenoids), *trans*-β-caryophyllene (sesquiterpenes), dill ether (terpene ethers), nonanal (aldehydes), myristicin (phenylpropanoids), bornyl acetate (esters), 1-decanol (alcohols), undecane (alkanes); the correlation coefficients (*R*^2^) for all compounds were >0.995, and results were expressed as mg·kg^−1^ fresh weight, fw.

### 2.4. Sensory Evaluation with a Trained Panel

A trained panel was used to evaluate the intensity of the main aroma attributes of fresh herbs. Samples were evaluated by seven panelists (five males and two females), with ages between 23 and 56 years old. Panelists belonged to the Food Quality and Safety research group of the Universidad Miguel Hernández de Elche and had over 1000 h of evaluation experience; they had been trained in descriptive evaluation of aromatic herbs [2,8,9].

An odor profile method was used to describe the dill samples. During two preliminary orientation sessions of 90 min, panelists discussed about the main odor characteristics of the herbs and agreed on their use of odor attributes. During these orientation experiments, panelists evaluated different coded samples of Spanish fresh aromatic herbs together with samples from field. Panelists agreed that the odor of the samples could be described using seven attributes: aromatic herb(dill/coriander/mint/parsley)-ID (clean fresh green, bitter, pungent aromatics associated with fresh dill/coriander/mint/parsley; reference: dill/coriander/mint/parsley water; preparation: 25 g chopped fresh dill/coriander/mint/parsley soaked in 300 mL room temperature deionized water for 15 min, filtered), green grass (green aromatics associated with newly cut-grass and leafy plants; reference: hexanal in propylene glycol, 10 g·L^−1^ = 6), citrus (aromatics associated with commonly known citrus fruits, such as lemons, limes, oranges, which could also contain a peely note; reference: McCormick lemon grass = 3.0; preparation: take 0.1 g of lemon grass and place it in a medium snifter together with 100 mL of deionized water, and cover it), pine (aromatics reminiscent of resinous pine tree; can be medicinal or disinfectant in character; reference: *El Corte Inglés* raw pine nuts = 3.0), spicy (sharp aromatics with a physically penetrating sensation in the nose reminiscent of radish and horseradish; reference: fresh radish = 3.0), earthy (humus-like aromatics that may or may not include damp soil, decaying vegetation or cellar-like characteristics; reference: hexanal in propylene glycol, 5 g·L^−1^ = 1.5), and woody (brown, musty aromatics associated with very fibrous plants and bark; reference: *Hacendado* dried parsley = 4.5). The key sensory descriptors (high intensities being related to high quality products) are: herb-ID, green grass, citrus, spicy, and pine; earthy and woody are not positively correlated with the herb quality. Reference products of these attributes with different intensity, were prepared and provided to the panel.

Individual booths with controlled illumination and temperature were used in this study. Three digit numbers were used to code samples, and they were randomly offered to panelists in plastic beakers of 100 mL with lids; samples were left 15 min at room temperature prior to analyses. The intensity of the seven odor attributes was scored using scale from 0 to 10, where 0 = none or not perceptible intensity, and 10 = extremely high intensity.

### 2.5. Statistical Analysis

To compare the experimental data two consecutive tests were performed: (i) one-way analysis of variance (ANOVA), and (ii) Tukey’s multiple range. Homogenous groups and the least significant difference (LSD) were determined at significance level of *p* ≤ 0.05. Statgraphics Plus 5.0 software (Manugistics, Inc., Rockville, MD, USA) was the program used for the statistical analyses.

## 3. Results and Discussion

### 3.1. Volatile Composition of Essential Oil of Dill

Dill was planted on 24 September 2014 and the first harvest date for commercial purpose was on 26 November 2014 (D1) while the second harvest date was on 11 February 2015 (D2). After isolation of the essential oil of dill shoots, 18 compounds were identified by GC-MS (Table 1). The identified volatile compounds can be grouped in eight main chemical groups: monoterpenes (110 compounds), followed by alkanes (two compounds), terpenoids (one compound), sesquiterpenes (one compound), aldehydes (one compound), phenylpropanoids (one compound), terpenes (one compound), and monoterpene ethers (one compound). The eight main compounds were: α-phellandrene, dill ether, β-phellandrene, limonene, *p*-cymene, α-pinene, *trans*-β-ocimene, and myristicin. In several studies, α-phellandrene was predominating in the “leaf” essential oil of dill from Romania, Egypt, and Finland [3,16,17,18]. The extraction of the essential oils can be done using different isolation techniques, and this analytical methodology can affect the volatile profile. For example, Huopalahti and Linko [3] found 22 compounds in dill by using a modified Soxhlet technique; however, α-phellandrene, dill ether, β-phellandrene were reported as the major compounds.

Table 2 shows that 3 compounds (α-phellandrene, dill ether, and β-phellandrene) clearly dominated the dill shoots essential oil, representing 85%–92% of the total concentration of volatile compounds. This experimental finding is supported as well by previous studies by Vokk *et al.* and Radulescu *et al.* [16,19] who reported that α-phellandrene, β-phellandrene, and dill ether were the main compounds of dill essential oil.

Volatile composition of dill essential oil at two commercial stages were investigated in the current study and the total concentration of volatile compounds was higher in commercial stage of D2 as compared to D1 stage; these trend was also true for the three main components (α-phellandrene, dill ether, and β-phellandrene). For α-phellandrene the concentration changed from 342 mg·kg^−1^ in D1 to 474 mg·kg^−1^ in D2 (an increase of 38.6 %), where the concentration in dill ether changed from 46.2 mg·kg^−1^ in D1 to 62.9 mg·kg^−1^ in D2 (an increase of 36.1 %) and from 46 mg·kg^−1^ (D1) to 60 mg·kg^−1^ (D2) for β-phellandrene (an increase of 30.4 %). Zlatev [20] and El-Gengaihi and Hornok [21] found that the essential oil content in dill increased continuously during the growing period. During the growth of dill, the contents of limonene, 3,6-dimethyl-2,3,3a,4,5,7a-hexahydrobenzofuran, and carvone increased, while those of α-phellandrene, β-pellandrene, myristicin, and apiol decreased. The total amount of aroma compounds varied widely during the growth [3].

### 3.2. Volatile Composition of Essential Oil of Parsley

Parsley was planted on 24 September 2014, the first harvest date for commercial purposes was on 19 November 2014 (P1), the second harvest date was on 5 January 2015 (P2), and the third harvest date was on 25 February 2015 (P3). Eighteen compounds were identified in the essential oil of parsley and the chemical classification of these 18 compounds was as follows: monoterpenes (nine compounds), sesquiterpenes (three compounds), terpenes (two compounds), monoterpenoids (one compound), monoterpene alcohol (one compound), esters (one compound), and phenylpropanoids (one compound). The main eight compounds of the essential oil of parsley shoots were: 1,3,8-p-menthatriene (38.4%–48.8%), β-phellandrene (22.2%–29.5%), myristcin (6.2%–11.1%), myrcene (5.8%–6.5%), terpinolene (4.2%–5.0%), limonene (2.7%–3.3%), α-pinene (1.6%–2.3%), and α-phellandrene (1.4%–2.7%). Vokk *et al.* [19] used Clevenger distillation method for essential oil isolation and gas chromatography for identifying the extracts and the major constituents of essential oil of parsley leaves were as following: myristicin (30.7%–42.7%), β-phellandrene (21.8%–35.9%), 1,3,8-p-menthatriene (5.4%–10.0%), and β-myrcene (4.5%–8.7%). Essential oils obtained by simultaneous distillation–extraction (SDE) from leaves of parsley plants and the main components were β-phellandrene, 1,3,8-p-menthatriene, α-p-dimethylstyrene, myristicin, β-myrcene, and apiole [15]. The results by Vokk *et al.* [19] and Petropoulos *et al.* [15] agree quite well with the results of this study.

Volatile composition of parsley essential oil at three commercial stages were investigated in the current study and the highest value of total concentration of essential oil was that of the commercial stage P1, 455 mg·kg^−1^, as compared to 414 and 418 mg·kg^−1^ at P2 and P3 stages, respectively. The main two components were 1,3,8-p-menthatriene and β-phellandrene, which represented 68%–72% of the total concentration of essential oil of parsley. 1,3,8-p-Menthatriene had the highest concentration, 222 mg·kg^−1^, at the P1 stage, while the highest concentration of β-phellandrene was found at 122 mg·kg^−1^ P2 harvest. For myristicin and myrcene, the highest concentration was in found at P2 stage, while for limonene was P3 and for terpinolene P1 (Table 3). For the essential oil of parsley plant Petropoulos *et al.* [15] recorded that, comparing the relative concentrations of the components for the two sowing dates, important differences were found for β-phellandrene (39.0%–22.0%) and 1,3,8-p-menthatriene (17.4%–45.7%) at the first growth stage and between 1,3,8-p-menthatriene (15.7%–29.0%) and myristicin (27.2%–4.6%) at the second stage.

### 3.3. Volatile Composition of Essential Oil of Coriander

The essential oil of three commercial samples of coriander was analyzed by GC-MS after extracted by hydrodistillation technique. The first crop (C1) was planted on 30 September 2014 and the harvest date was 19 November 2014, the second crop (C2) was planted on 22 October 2014 and harvested on 5 January 2015 and the third crop (C3) was planted on 29 December 2014 and harvested on 25 February 2015.

GC-MS was used to identify the chemical composition of essential oil of coriander plant. Twenty-four compounds were identified (Table 1) and the chemical classification of these 24 compounds were as follows: aldehydes (11 compounds), followed by alcohols (five compounds), esters (two compounds), alkanes (two compounds), monoterpenes (two compounds), terpenoids (one compound), and terpenes alcohol (one compound). The main seven compounds of the essential oil of coriander shoots were: decanal (30.7 mg·kg^−1^, mean of all treatments), *E*-2-dodecenal (mean of 26.9 mg·kg^−1^), dodecanal (22.0 mg·kg^−1^), octane (18.6 mg·kg^−1^), 1-decanol (5.13 mg·kg^−1^), undecanal (4.30 mg·kg^−1^), and *E*-2-tridecenal (3.54 mg·kg^−1^). Given the results in Table 4, decanal, *E*-2-dodecenal, dodecanal, and octane represented a big percentage of the total concentration of volatile compounds in the essential oil of coriander shoots (70.9%–82.5%). The highest concentrations of *E*-2-dodecenal (39.9 mg·kg^−1^), dodecanal (24.4 mg·kg^−1^), and octane (20.7 mg·kg^−1^) were found at C2 harvest, while the maximum value of decanal was at C3 with 36.4 mg·kg^−1^ (Table 4). Nurzyńska-Wierdak [22] reported that the essential oil of the coriander herb contained the highest amount of aliphatic aldehydes, with decanal, *E*-2-dodecanol, and *E*-2-decenol having the highest contents. In coriander (*Coriandrum sativum* L.) the most abundant compounds are *E*-2-decenal, *E*-2-dodecenal, decanal, dodecanal, *E*-2-tridecenal and tetradecenal; these compounds have characteristic green, soapy, and cilantro-like aromas and are particularly important in the overall aroma of the *C. sativum* herb [23,24]. These results are in concordance to a large extent with the current results.

### 3.4. Volatile Composition of Essential Oil of Mint

Peppermint (*Mentha piperita* L.) was planted on 6 October 2014 and the first harvest date was on 11 December 2014 (M1) while the second harvest date was on 5 February 2015 (M2). After isolation of the essential oil of mint shoots, 27 compounds were identified by GC-MS (Table 1). The identified volatile compounds can be grouped in eight main chemical groups: monoterpenes (six compounds), terpenoid alcohols (three compounds), sesquiterpenes (two compounds), aldehydes (one compound), terpenes (three compounds), esters (two compounds), aldehyde (one compound), terpene alcohols (one compound), and polycyclic alkenes (one compound). The eight main compounds were: carvone, limonene, *cis*-carveol, *trans*-sabinene hydrate, *trans*-caryophyllene, myrcene, santene, and *trans*-β-ocimene.

Volatile composition of mint essential oil at two commercial stages was investigated in the current study and the total concentration of volatile compounds in the essential oil was higher in plants of the commercial stage M1 as compared to those of M2. The contents of the main compound, carvone, also followed this trend (M1 > M2), while the concentrations of limonene and *cis*-carveol were higher in M2 as compared to M1. For carvone the concentration decreased from 2462 mg·kg^−1^ in M1 to 1854 mg·kg^−1^ in (M2) (a decrease of 24.7 %), while the concentration of limonene increased from 590 mg·kg^−1^ in M1 to 735 mg·kg^−1^ in M2 (an increase of 24.6 %) (Table 5).

According the previous studies, the harvest date and harvest time can affect the water content, the concentrations of menthol and menthofuran and the yield of limonene, menthol, and menthofuran in Mentha canadensis [25,26]. Besides, in Japanese mint (*Mentha arvensis* L.) the content of menthol was not affected by the planting date or harvesting schedule but menthone signficantly decreased with the delay in harvesting [27].

The major components of peppermint essential oil were menthol (30.35 %), menthone (21.12 %), and trans-carane (10.99 %) according to previous studies [5]. These results did not agree with the results obtained in the current study. *Mentha piperita* (peppermint) showed in the composition of its essential oil a higher content of monoterpenes D-carvone (58.79 %) and limonene (28.29 %) [28]. Rohloff [29] found increased levels of other oxygenated monoterpenes and limonene in their work compared to *Mentha piperita* grown in Norway [28]. These results correspond to a large extent with the results in the current study.

### 3.5. Descriptive Sensory Evaluation

Volatiles directly affect the sensory quality of fresh fruits, vegetables and aromatic herbs. Within the sensory quality, the odor (perception of volatile compounds with the food outside the mouth) [30] plays an important role, especially in essential oils of aromatic herbs; the aroma is formed by a complex group of chemical substances, which includes aldehydes, alcohols, ketones, esters, lactones, terpenes, among other volatile compounds. The concentration of these volatile compounds is generally low (mg· kg^-1^) and can be affected by a number of agronomic (variety, climatological conditions, ripening stage) [31,32] and technological (harvest, post-harvest treatments, storage and processing conditions) factors [33]. The quality of the vegetal products can be affected by both too high or too low concentrations of the volatile compounds; thus an equilibrium among them is necessary. Thus, all the information related to descriptive sensory evaluation (DSA), which is going to be shown, corresponds to those samples with the highest concentrations of the volatile compounds present in each essential oil (Table 2, Table 3, Table 4 and Table 5); of course, this is a first step that will need further research but it is a very important step, which is done by the first time and will provide very practical information for farmers. According to this statement, the DSA was carried out with the following samples: D2 (dill harvested at the second commercial stage); P1 (parsley harvested at the first commercial stage); C2 (coriander harvested at the second commercial stage); and M1 (mint harvested at the third commercial stage).

**Figure 1:**
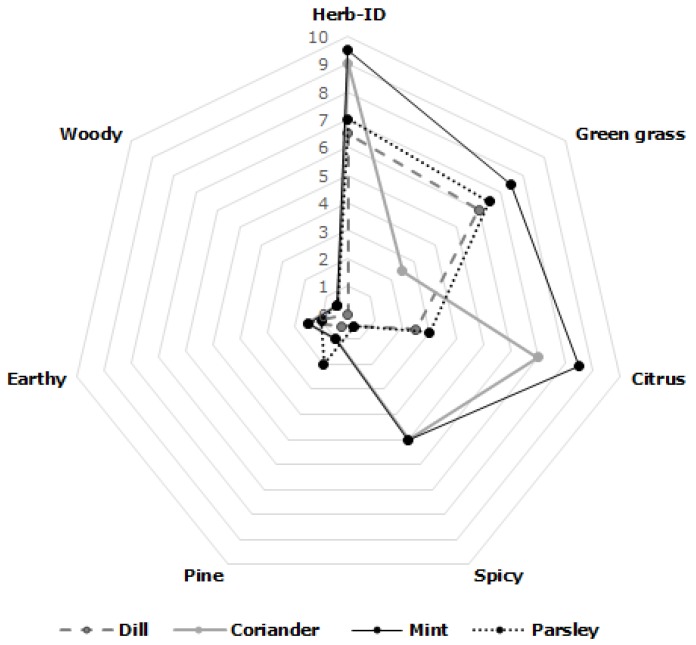
Descriptive sensory analysis of Spanish aromatic herbs.

Figure 1 shows the DSA profiles of different aromatic herb samples. The descriptors selected for the DSA were successfully used by this research group in previous studies [2,8,9].

Dill samples (D2), with a total number of 18 volatile compounds accounting a total concentration of 649 mg·kg^−1^ of its essential oil, were characterized by high intensity of dill-ID (herb-ID in Figure 1) (6.5), green grass (6.0), and citrus (2.5) notes (Figure 1). On the other hand, dill samples scored low values of attributes such as spicy (0.5), earthy (1.5), pine (0.5), or woody (0) (Figure 1).

Parsley samples (P1), with total number of 18 volatile compounds accounting a total concentration of 455 mg·kg^−1^ of its essential oil, were characterized by high intensity of parsley-ID (herb-ID in Figure 1) (7), citrus (3), and green grass (6.5) notes (Figure 1), while undesirable parsley attributes scored low values, for instance spicy (0.5), earthy (1.0), pine (2.0), or woody (0.5) (Figure 1).

Coriander samples (C2), with a total number of 24 volatile compounds accounting a total concentration of 134 mg·kg^−1^ of its essential oil, were characterized by high intensity of coriander-ID (herb-ID in Figure 1) (9), citrus (7), and spicy (5) and low values of green grass (2.5), earthy (1.5), pine (1.0), or woody (0.5) (Figure 1).

Finally, mint samples (M1), with the highest number of volatile compounds (27) and the higher concentration of these compounds in its essential oils (3326 mg·kg^−1^), were characterized by high intensity of mint-ID (herb-ID in Figure 1) (9.5), green grass (7.5), citrus (8.5), and spicy (5).

The present study was the first one reporting data on descriptive sensory analysis of aromatic herbs at their optimal harvest time according to the content of volatile compounds of their essential oils. This information is valuable for farmers because the reported data shows the optimal date according to the highest productions of essential oils and high sensory quality.

## 4. Conclusions

Considering all the data generated in this study, the final recommendation according to the essential oil content and sensory quality is to harvest at the following dates: for dill (11 February 2015) however, for parsley (19 November 2014) while, for coriander (5 January 2015), and for mint (11 December 2014), there are relevant aspects which must subjected to further studies such as, different irrigation treatments, different plant densities, or fertilization conditions. In addition, the effect of pre-harvest treatments with organic compounds may be employed.

## Figures and Tables

**Table 1:** Identification of essential oils found in dill, parsley, coriander, and mint samples.

Compound	Herb	RT (min)	Retention Indexes (RI)		Descriptor ^¶^
			Exp. ^†^	Lit. ^†^	
*trans*-2-Hexenal ^‡^	dill	11.09	806	800	Green, banana, aldehydic ^‡^
Octane	coriander	12.12	808	800	
α-Thujene	dill	13.19	873	905	Woody, green, herb
Santene	mint	13.29	879	880	
α-Pinene	dill, parsley, mint	13.58	896	909	Fresh, camphor, sweet, pine, earthy, woody
Camphene	mint	14.38	944	945	Fresh, woody, fir, terpene
Sabinene	dill, parsley	14.89	975	975	Woody, terpene, citrus, pine, spice
Myrcene	dill, parsley, mint	15.15	991	991	Peppery, terpene, spicy
β-Pinene	dill, parsley	15.25	997	990	Dry, woody, pine, hay, green
*cis*-3-Hexenyl acetate	parsley, coriander, mint	15.70	1008	1009	Fresh, green, sweet, fruity, banana, apple
α-Phellandrene	dill, parsley	16.20	1020	1013	Citrus, herbal, terpene, green, woody, peppery
α-Terpinene	mint	16.68	1031	1018	Woody, terpene, lemon, herbal, citrus
*p*-Cymene	dill, parsley, mint	16.88	1036	1034	Fresh, citrus, terpene, woody, spice
Limonene	dill, parsley, coriander, mint	17.08	1040	1039	Terpene, pine, herbal, peppery
β-Phellandrene	dill, parsley, coriander	17.25	1044	1036	Mint, terpentine
*trans*-β-Ocimene	dill, parsley, mint	17.38	1047	1047	Citrus, tropical, green, terpene, woody
γ-Terpinene	parsley, mint	18.20	1066	1066	Woody, terpene, lemon, lime, tropical, herbal
*trans*-Sabinene hydrate	mint	19.00	1084	1087	Warm, balsamic, woody
Terpinolene	dill, parsley	19.47	1095	1097	Fresh, woody, sweet, pine, citrus.
Undecane	dill, coriander	19.63	1098	1099	Fusel-like
Linalool	coriander, mint	19.82	1103	1103	Citrus, orange, floral, terpy, rose
Nonanal	coriander, mint	20.03	1107	1107	Aldehydic, rose, fresh, orris, orange, peel
1,3,8-*p*-Menthatriene	parsley	20.74	1125	1115	Turpentine, camphor, herbal, woody
*cis*-Limonene oxide	mint	21.87	1149	1140	Fresh, citrus
*trans*-Limonene oxide	mint	22.04	1153	1147	Fresh, citrus, mild, green
*cis*-*p*-Mentha-2.8-dien-1-ol	mint	23.74	1192	1193	
*trans*-*p*-Mentha-2.8-dien-1-ol	mint	24.07	1199	1196	Fresh, minty
Dill ether	dill	24.40	1206	1187	Herbal, dill, spicy
α-Terpineol	parsley	24.74	1213	1200	Pine, terpene, lilac, citrus, woody, floral
Decanal	coriander	24.79	1214	1207	Sweet, aldehydic, orange, waxy, citrus rind
*cis*-Carveol	mint	25.05	1220	1221	Caraway, spicy, citrus, fruity
*trans*-Carveol	mint	25.43	1228	1217	Caraway, green, oily
Carvone	dill, coriander, mint	27.13	1264	1262	Herbaceous, grapefruit, pepper, spicy, woody
*E*-2-Decenal	coriander	27.54	1273	1278	Earthy, coriander green, mushroom, aldehydic
1-Decanol	coriander	28.45	1292	1287	Floral, orange, sweet, clean watery
Tridecane	dill	29.06	1297	1299	Citrus, fruity, Fusel-like
Bornyl acetate	mint	29.10	1305	1291	Woody, camphor, mentholic, spicy
Undecanal	coriander	29.63	1317	1310	Fresh, citrus, waxy, aldehydic
Carvomenthyl acetate	mint	30.70	1339	1344	
*E*-2-Undecenal	coriander	32.40	1375	1371	Aldehydic, citrus
1-Undecanol	coriander	33.88	1407	1386	Earthy, soapy, waxy, fatty, honey, coconut
β-Bourbonene	mint	34.08	1412	1407	Herbal, Woody
Decyl acetate	coriander	34.13	1412	1410	Waxy, sweet, fatty, creamy
β-Caryophyllene	mint	34.26	1416	1418	Sweet, woody, spice clove dry
Dodecanal	coriander	34.39	1419	1420	Orange, fatty, herbaceous
*trans*-β-Caryophyllene	parsley, mint	35.68	1448	1455	Woody, spicy
*Z*-2-Dodecenal	coriander	36.37	1463	1467	Green, citrus, fruity, mandarin orange, herbal
*E*-2-Dodecenal	coriander	37.12	1480	1468	Citrus, mandarin orange, aldehydic
α-Humulene	mint	37.48	1489	1489	
*E*-2-Dodecen-1-ol	coriander	37.78	1495	1483	Oily, fatty
1-Dodecanol	coriander	38.08	1502	1485	Earthy, soapy, waxy, fatty, honey, coconut
Germacrene-D	dill, parsley, mint	38.42	1475	1477	Woody, spice
Tridecanal	coriander	38.96	1522	1518	Fresh, aldehydic, citrus, grapefruit peel
Nerolidol	parsley	38.97	1525	1528	Floral, green, citrus, woody
Myristicin	dill, parsley	39.97	1543	1532	Spice, warm, balsam, woody
*E*-2-Tridecenal	coriander	41.58	1582	1571	Citrus, peel tangerine
1-Tetradecanol	coriander	42.81	1615	1618	Fruity, coconut
Tetradecanal	coriander	43.31	1632	1623	Dairy, creamy, fishy with a fruity, pear nuance.

^†^ RT = retention time; Exp. = experimental and Lit. = Literature; ^‡^ All compounds were identified using retention indexes, mass spectra and retention time of standards; ^¶^ SAFC (2015); www.pherobase.com; www.thegoodscentscompany.com.

**Table 2:** Volatile composition of dill essential oil at two commercial stages (mg·kg^−1^·fw).

Compound	ANOVA ^†^	D1	D2
		Concentration, (mg·kg^−1^·fw)	
*trans*-2-Hexenal	***	0.18 b ^¥^	1.81 a
α-Thujene	***	1.35 b	1.81 a
α-Pinene	***	7.84 b	8.70 a
Sabinene	***	0.35 b	0.51 a
Myrcene	***	2.41 b	3.20 a
β-Pinene	***	0.66 a	0.31 b
α-Phellandrene	***	342 b	474 a
*p*-Cymene	***	12.5 a	3.92 b
Limonene	***	17.6 b	21.4 a
β-Phellandrene	***	46.0 b	60.0 a
*trans*-β-Ocimene	***	5.13 b	7.50 a
Terpinolene	***	2.53 a	0.33 b
Undecane	***	4.38 a	1.11 b
Dill ether	***	46.2 b	62.9 a
Carvone	NS	0.02 a	0.02 a
Tridecane	***	0.56 a	0.20 b
Germacrene-D	***	4.19 a	1.38 b
Myristicin	***	13.8 a	0.02 b
TOTAL	***	508 b	649 a

^†^ NS = not significant *F* ratio (*p* < 0.05); *** significant at *p* < 0.001. ^‡^ Treatment means of the ANOVA test (values are the mean value of 3 replications). ^¥^ Values followed by the same letter, within the same row, were not significant different (*p* < 0.05), Tukey’s multiple-range test.

**Table 3:** Volatile composition of parsley essential oil at three commercial stages (mg·kg^−1^·fw).

Compound	ANOVA ^†^	P1	P2	P3
		Concentration, (mg·kg^−1^·fw)		
α-Pinene	***	7.07 c ^¥^	8.53 b	9.65 a
Sabinene	***	0.30 b	0.38 b	0.55 a
Myrcene	***	27.0 a	27.1 a	24.3 b
β-Pinene	***	2.47 c	4.14 a	3.64 b
*cis*-3-Hexenyl acetate	***	1.73 a	0.35 b	0.33 b
α-Phellandrene	***	6.46 c	11.0 a	8.63 b
*p*-Cymene	***	1.40 b	1.41 b	1.83 a
Limonene	***	12.5 b	11.3 b	13.7 a
β-Phellandrene	***	101 c	122 a	110 b
*Trans*-β-Ocimene	***	2.89 b	2.33 c	3.69 a
γ-Terpinene	***	0.45 a	0.29 b	0.31 b
Terpinolene	***	22.8 a	17.2 b	18.5 b
1,3,8-*p*-Menthatriene	***	222 a	159 c	192 b
α-Terpineol	***	0.26 c	0.40 b	0.82 a
*trans*-β-Caryophyllene	***	0.61 c	1.64 b	2.15 a
Germacrene-D	***	0.96 c	1.63 a	1.39 b
Nerolidol	***	0.15 b	0.07 c	0.23 a
Myristcin	***	45.1 a	45.9 a	25.9 b
TOTAL	***	455 a	414 b	418 b

^†^ NS = not significant F ratio (*p* < 0.05); *** significant at *p* < 0.001. ^‡^ Treatment means of the ANOVA test (values are the mean value of 3 replications). ^¥^ Values followed by the same letter, within the same row, were not significant different (*p* < 0.05), Tukey’s multiple-range test.

**Table 4:** Volatile composition of coriander essential oil at three commercial stages (mg·kg^−1^·fw).

Compound	ANOVA ^†^	C1	C2	C3
		Concentration, (mg·kg^−1^·fw)		
*Octane*	***	16.9 c ^¥^	20.7 a	18.2 b
*cis*-3-Hexenyl acetate	***	0.64 b	1.17 a	1.11 a
Limonene	***	1.06 a	0.18 b	0.00 c
β-Phellandrene	NS	0.04 c	0.14 b	1.39 a
Undecane	***	0.45 b	1.09 a	0.36 b
Linalool	***	0.06 a	0.14 a	0.05 a
Nonanal	NS	0.01 c	0.92 a	0.22 b
Decanal	***	30.3 b	25.5 c	36.4 a
Carvone	***	3.06 a	0.01 c	0.37 b
*E*-2-Decenal	***	0.27 b	0.39 b	1.47 a
1-Decanol	***	3.70 c	5.05 b	6.64 a
Undecanal	***	2.23 c	3.93 b	6.70 a
*E*-2-Undecenal	NS	0.01 c	0.44 b	0.75 a
1-Undecanol	NS	0.05 b	0.11 a	0.05 b
Decyl acetate	NS	0.01 a	0.03 a	0.00 a
Dodecanal	***	24.1 a	24.4 a	17.6 b
*Z*-2-Dodecenal	***	0.12 b	0.26 a	0.10 b
*E*-2-Dodecenal	***	15.0 c	39.9 a	25.7 b
*E*-2-Dodecen-1-ol	***	1.90 a	1.00 b	0.04 c
1-Dodecanol	***	1.80 a	0.21 b	0.01 c
Tridecanal	***	1.90 a	1.18 b	1.22 b
*E*-2-Tridecenal	***	1.33 b	4.69 a	4.59 a
1-Tetradecanol	***	0.12 b	0.27 a	0.08 b
Tetradecanal	***	1.61 b	1.98 a	0.88 c
TOTAL	***	107 c	134 a	124 b

^†^ NS = not significant F ratio (*p* < 0.05); *** significant at *p* < 0.001. ^‡^ Treatment means of the ANOVA test (values are the mean value of 3 replications). ^¥^ Values followed by the same letter, within the same row, were not significant different (*p* < 0.05), Tukey’s multiple-range test.

**Table 5:** Volatile composition of mint essential oil at two commercial stages (mg·kg^−1^·fw).

Compound	ANOVA ^†^	M1	M2
		Concentration, (mg·kg^−1^·fw)	
Santene	***	22.5 a ^¥^	24.05 a
Camphene	NS	2.17 b	3.50 a
β-Pinene	***	12.3 a	13.1 a
Myrcene	***	23.6 b	28.3 a
*cis*-3-Hexenyl acetate	NS	0.56 a	0.62 a
*p*-Cymene	NS	1.29 b	2.55 a
α-Terpinene	NS	0.54 b	2.90 a
Limonene	***	590 b	735 a
*trans*-β-Ocimene	***	19.1 a	18.2 a
γ-Terpinene	NS	1.31 b	5.17 a
*trans*-Sabinene hydrate	***	34.3 b	73.6 a
Nonanal	***	10.6 a	12.0 a
Linalool	NS	1.29 b	2.23 a
*cis*-Limonene oxide	NS	1.28 a	1.23 a
*trans*-Limonene oxide	NS	2.84 a	1.79 b
*cis*-*p*-Mentha-2,8-dien-1-ol	NS	6.43 a	6.39 a
*trans*-*p*-Mentha-2,8-dien-1-ol	NS	4.45 b	11.4 a
*cis*-Carveol	***	65.3 b	85.8 a
*trans*-Carveol	NS	8.12 a	9.73 a
Carvone	***	2462 a	1854 b
Bornyl acetate	NS	0.51 a	0.43 a
Carvomenthyl acetate	***	12.0 b	14.7 a
β-Bourbonene	***	14.6 a	14.0 a
β-Caryophyllene	NS	2.30 b	3.59 a
*trans*-Caryophyllene	***	22.1 b	35.5 a
Alloaromadendrene	NS	1.74 b	2.57 a
α-Humulene	NS	3.58 b	5.11 a
TOTAL	***	3326 a	2968 b

^†^ NS = not significant F ratio (*p* < 0.05); *** significant at *p* < 0.001. ^‡^ Treatment means of the ANOVA test (values are the mean value of 3 replications). ^¥^ Values followed by the same letter, within the same row, were not significant different (*p* < 0.05), Tukey’s multiple-range test.
